# Major Histocompatibility Complex-B haplotype and ovarian graft response

**DOI:** 10.1016/j.psj.2023.102850

**Published:** 2023-06-09

**Authors:** Chi Cuong Quach, Janet E. Fulton, James D. Benson, Pamela Walker, Crissandra Auckland, Carl Lessard

**Affiliations:** ⁎ Western College of Veterinary Medicine, University of Saskatchewan, Saskatoon, SK, S7N 5B4, Canada; † Agriculture and Agri-Food Canada, Saskatoon, SK, S7N 0×2, Canada; ‡ Hy-Line International, Dallas Center, IA, 50063, USA; § Department of Biology, College of Art and Science, University of Saskatchewan, Saskatoon, SK, S7N 5A5, Canada

**Keywords:** immunosuppression, MHC-B, ovary, gonadal transfer, vitrification

## Abstract

Gonadal tissue transfer is considered one of the best methods to preserve genetic variability. Poultry hosts can receive a gonad from a donor of a different genetic background, sustain the growth of this graft, and produce gametes from it. Unfortunately, the host's strong immune response may significantly reduce the gonadal graft's ability to reach maturity. Our study aimed to evaluate the influence of MHC-B alleles in rejecting a gonadal graft of similar or different genetic backgrounds. In the first experiment, ovarian tissue was transplanted to chicks of similar genetic backgrounds, either Lohmann White (**LW**) with variable MHC-B or Barred Rock (**BR**) with fixed MHC-B. The sustained growth of donor ovarian tissues occurred in (4/7 hosts) BR (MHC-B matched) hosts only—one of these graft-positive-BR hens produced eggs derived from the donor ovary. No grafts were recovered when the host and the donor had an LW background (0/9; MHC-B mismatched). In the second experiment, ovarian transplantation was done between chicks of either similar or different genetic backgrounds (Brown Leghorn [**BL**], BR, and BL/BR F1). The 2 pure lines contained only one MHC-B allele, whereas the F1 heterozygotes had both. All host birds were given a daily dose of an immunosuppressant (mycophenolate mofetil) until maturity. The success rate was assessed by microsatellite genotype confirmation of donor-derived ovaries plus physiological and histological analyses of ovarian grafts. In this second experiment, 11 out of 43 ovarian hosts laid eggs. However, all fertilized eggs from these hens were derived from the remnant host ovarian tissue, not from the donor ovaries. A necropsy assessment was done on all 43 host birds. Ten donor grafts were recovered from hosts having matched (6 hosts) and mismatched (4 hosts) MHC-B, and none were functional. Interestingly, 6 of them were enclosed by a serous membrane capsule filled with fluid and had various tissue growth. In addition, clusters of immune cells were observed in all recovered donor grafts. Our results demonstrated that genetic background could greatly influence the success of gonadal transfer in chickens.

## INTRODUCTION

Heritage breeds represent a source of genetic diversity and are well adapted to diverse circumstances, including extreme environments, variable feed quality, and multiple pathogen challenges. Unfortunately, the number of heritage chicken breeds has been decreasing over the last 4 decades with the development of highly productive and efficient commercial production varieties that meet the high demand of consumers for low-cost chicken meat and eggs (Pisenti et al., 1999; Fulton and Delany, 2003). Various methods have been proposed to maintain these valuable heritage chicken breeds, each with advantages and disadvantages. In-situ preservation of poultry lines is laborious and costly, and does not protect against flock loss due to challenges by highly infectious diseases nor does it protect against genetic bottle-necks due to small population size (Santiago-Moreno and Blesbois, 2022). Long-term storage of sperm cells in liquid nitrogen is cost efficient, and this form of germplasm can be preserved for many years; however, it requires many collections from a large number of roosters to include the genetic diversity of each breed. Furthermore, the fertilization ability of frozen/thawed semen can vary considerably between lines (Long et al., 2010). Also, males are the homogametic sex; thus they do not include the W chromosome and mitochondrial DNA derived from the female gametes. Therefore, semen conservation cannot preserve all of the genetic variations within a heritage line. Cryopreservation of the female gamete has been unsuccessful for avian species due to the large egg size. Primordial germ cells can be collected and stored, but many fertilized eggs are required to obtain a sufficient number of cells for the preservation of the genetic variability within the line, which can be a limitation for heritage breeds (Nakamura, 2016). Propagation of PGC has been demonstrated and shown to be capable of germline transmission (Woodcock et al., 2019; Lazar et al., 2021), but it requires long period of culture and access to a sterile poultry line. However, it offers the possibility of transferring propagated PGCs from multiple sources into a single host (Ballantyne et al., 2021). Alternative strategies have been proposed to solve the issues related to preserving heritage breeds, such as preserving both male and female gonadal tissues (Song and Silversides, 2006; Song and Silversides, 2007a).

Vitrification (ice-free preservation in an amorphous glassy state) of gonadal tissue has been successful in many species, including poultry (Liu et al., 2012a). If testes or ovaries from poultry breeds could be preserved in liquid nitrogen, they could be transferred to a host of different genetic backgrounds to regenerate the breed when needed (Song and Silversides, 2006). However, the success of gonadal transfer varies considerably between poultry breeds (Kosenko, 2007; Liptoi et al., 2013, 2020). In the aforementioned citations, many gonadal hosts either did not have the presence of a gonadal graft, had degenerated gonadal tissue, or had encapsulated ovarian tissue. Histocompatibility barriers with subsequent immunological rejection were proposed as the main factor affecting graft loss (Kosenko, 2007). Genetic compatibility was suggested as an important factor in increasing the chance of sustaining the growth of a gonadal graft in a host and minimizing a host graft rejection response (Liptoi et al., 2013, 2020).

The host's immune response is mediated by genes within the major histocompatibility complex-B (**MHC-B**), which is associated with resistance to many poultry diseases (Fulton, 2020). Differences in the MHC-B region are also identified as the genetic basis for tissue transplantation rejection (Schierman and Nordskog, 1961). This cluster of genes is highly polymorphic in chickens, with over 90 haplotypes identified, many of which are breed-specific (Fulton, 2017; Iglesias et al., 2019; Berres et al., 2020; Fulton, 2020; Manjula et al., 2021). Heritage breeds maintain much diversity of MHC-B haplotypes (Fulton et al., 2016), suggesting that noncommercial breeds are likely a good reservoir of genetic diversity. Interestingly, the Barred Plymouth Rock line (**BR**) maintained at the University of Saskatchewan was found to contain 2 MHC-B types, with one being at a very high frequency. Also, the Dark Brown Leghorn line maintained at the University of Alberta was found to be fixed for a different MHC-B type (Fulton et al., 2016). This fortuitous discovery provided the resources to examine the impact of MHC-B differences on gonadal tissue transplantation and rejection. Our study focused on transferring fresh or vitrified-warmed ovarian tissues to females to provide information on preserving poultry ovarian gonads of heritage breeds.

## MATERIALS AND METHODS

### Chick Breed Preparation and Incubation Procedures

Barred Plymouth Rock and Lohmann White Leghorn (**LW**) fertilized eggs were obtained from the Poultry Research and Teaching Unit at the University of Saskatchewan (Saskatoon, Canada). Dark Brown Leghorn (**BL**) fertilized eggs were purchased from the Poultry Research Center at the University of Alberta (Edmonton, Canada). Two MHC-B haplotypes (BSNP-B02 (freq. = 0.06) and BSNP-P04 (freq. = 0.94) are found in the BR flock, and the BL flock is fixed for 1 MHC-B haplotype (BSNP-O01; Fulton et al., 2016). The LW flock was a commercial White Leghorn production bird with 2 MHC-B alleles as indicated by the MHC-specific LEI0258 genotype. Hens were artificially inseminated using a pool of semen manually collected from roosters of the same source flock to produce LW, BR, or BL chicks. Barred Plymouth Rock hens and roosters produced during this study did not show the presence of the low-frequency MHC-B allele (BSNP-B02) when genotyped with LEI0258 (data not shown). BL/BR F_1_ birds were produced by artificial insemination of BL hens with pooled BR semen and maintained at the University of Saskatchewan Animal Care Unit of the University of Saskatchewan. All fertilized eggs were incubated (Digital 1502 Sportsman, GQF Manufacturing, Savannah, GA, USA) at 99.9°F and 60% relative humidity for 18 d and then transferred to a hatcher (Digital 1550 Sportsman; GQF Manufacturing) at 98°F and 70% relative humidity for the last 3 d of 21 d incubation period. At hatching, chicks were used as either the source (donor) of gonadal tissue or as a host of fresh or vitrified-warmed ovaries. The Animal Research Ethics Board of the University of Saskatchewan approved bird handling procedures for this study (UAP #20120110).

### DNA Extraction and Genotyping of Grafted Tissues

A small piece of tissue was taken from all birds used in this study. When a graft was recovered, it was genotyped with a tissue sample of both donor and host to determine whether the recovered graft was donor- or host-derived. Genomic DNA was isolated using the DNeasy Blood and Tissue kit (Qiagen Inc., Burlington, Ontario, Canada; cat#69506). Eleven highly variable microsatellite loci were used to differentiate donor and host tissues (ADL0278, LEI0094, MCW0216, MCW0081, MCW0034, MCW0016, LEI0166, LEI0258, MCW0222, MCWO295, MCW0037) on an ABI 3500xL (Applied Biosystems) DNA sequencer. The microsatellite LEI0258 was used specifically to determine the MHC type for the donor, host, and graft (Fulton et al., 2006). Genotypes were determined using GeneMapper version 5.0 software (Applied Biosystems).

### Collection of Fresh and Vitrified-Warm Ovarian Tissue for Transplantation

For fresh ovaries, 1- to 3-day-old chicks from each breed were humanely euthanized by cervical dislocation. Ovaries were harvested from the abdominal cavity, and any remnants of connective tissue or blood clots were removed. The collected ovaries were placed in a holding medium (**HM**) composed of Dulbecco's phosphate-buffered saline solution and 20% fetal bovine serum and maintained on ice to store before the procedure. Unless otherwise stated, all chemicals and reagents were purchased from Life Technologies Inc. (Burlington, Canada).

An adapted and modified version of the vitrification warming protocol described by Liu and colleagues (Liu et al., 2012b) was followed for the vitrified-warmed ovaries. The harvested and cleaned ovaries were gently placed on acupuncture needles (0.20 mm × 30 mm, Dong Bang Acupuncture Inc., Chungnam, Korea), then submerged in the first equilibrium solution held on ice (7.5% dimethyl sulfoxide (**DMSO**), 7.5% ethylene glycol (**EG**; cat#102466) mixed in HM for 15 min (modification of Liu's protocol based on preliminary data to optimize vitrification; data not published). In the second step, the ovaries were transferred to the next equilibrium solution (15% DMSO, 15% EG, 0.5M sucrose mixed in HM) for 3 min, also on ice. Afterward, the ovaries were blotted on gauze to remove excess moisture and immediately plunged into a tray holding liquid nitrogen (**LN_2_**). The frozen tissues were transferred into precooled cryovials in LN vapor environment and kept in a tank with LN_2_ for at least 5 d before the warming process prior to transplantation.

For the warming procedure, the cryovials containing the ovarian tissue were removed from the LN_2_ storage tank, and the needles with the gonadal tissue were quickly removed from the cryovial and placed into a weigh boat filled with LN_2_. Following this step, the ovaries were immediately transferred into HM with added 1 M sucrose (cat# S1888) at room temperature (**RT**) for 5 min. The needles and tissue were then transferred to a 0.5 mol/L sucrose solution in HM at RT for 5 min and finally moved to 0.25 M sucrose in HM at RT for 5 min. In the last step, the ovaries were placed in a petri dish containing DMEM (Dulbecco's Modified Eagle Medium) with 20% FBS on ice, and the tissues were removed from the needle until they were grafted. The time from postwarming to tissue implantation was a maximum of 3 h to prevent damage.

### Sex Identification of Host Chicks

A PCR-based assay was used to identify the sex of each chick before the surgical procedures (Boutette et al., 2002). Only female chicks were selected for this study to avoid any sex incompatibilities. Blood from newly hatched chicks was collected from the brachial wing vein and deposited on FTA cards (Qiagen, Radnor, PA). DNA was extracted from the FTA card using the standard protocol, and 1 µl of DNA extract was used for the PCR. The primers used to amplify CHD-Z genes were F 5′-TCTGGATCGCTAAATCCTTT-3′ and R 5′-CTCCCAAGGATGAGRAAYTG-3′. The forward primer was labeled with 6-FAM to allow detection of the amplicon on the ABI Genetic Analyzer 3500xL (Applied Biosystems). The CHD-Z gene is known to produce different lengths of PCR amplicons in male vs. female chickens and thus is used to determine sex (Vucicevic et al., 2013).

### Experimental Design

Two experiments were conducted by orthotopic transplantation, in which the donor ovary is removed and transferred to the same position in the host. Pullets were raised until maturity and artificially inseminated with a pool of semen from roosters of similar genetic backgrounds to produce fertilized eggs. For each experiment, a group of control birds without surgery was kept until maturity for the weight, maturation, and laying time.

For the first experiment, we tested our ability to transfer fresh or vitrified-warmed gonads into a host of similar genetic backgrounds, as described by Song and Silversides (2007b). Prior to birds being used in surgery, both the BR and the LW lines were pregenotyped to determine alleles present in each population, allowing segregation of both private and fixed alleles in each respective population. In addition, we ensured that the LW donor and host did not have the same MHC-B haplotype, whereas all BR donors and hosts were MHC-B matched. Fresh or vitrified-warmed ovarian tissues from 1- to 3-day-old BR and LW were transplanted to hosts of the same breed and age. The number of LW and BR hosts was 9 and 7, respectively. Sixteen donor ovaries were used (4 fresh LW ovaries, 5 vitrified-warmed LW ovaries, 4 fresh BR ovaries, and 3 vitrified-warmed BR ovaries). The control group included chicks without surgery (*n* = 3).

This second trial had a 2-fold purpose: to evaluate the potential of different host chicken breeds to maintain transplanted ovarian tissue and to assess the role of MHC-B in tissue transplantation. A 3 × 3 experiment was designed with the MHC-B types, requiring a total of 9 donor and host combinations. The number of birds for each combination is indicated in Table 2. Prior to birds being used in surgery, both the BR and the BL lines used in this experiment were genotyped to determine alleles present in each population, allowing segregation of both private and fixed alleles in each respective population.

### Transplantation Procedure

Surgical procedures were performed as described in the protocol according to Song and Silversides and Liptoi et al. (Song and Silversides, 2007b, Song and Silversides, 2008; Liptoi et al., 2023) with minor modifications. Briefly, the host chicks were weighed and orally administrated 0.5 mg/kg of Meloxicam to provide pre-emptive analgesia (Metacam, Boehringer Ingelheim Ltd., Burlington, Canada) and then anesthetized by Isoflurane 99.9% (Fresenius Kabi Animal Health, Richmond Hill, Canada). Once anesthetized, a modified mask was attached to the chick's head to facilitate the nasal inhalation of the anesthetic. The surgical area was shaved and cleaned with Germi-Stat Gel 4% (Germiphene Corporation, Branford, Canada). The chicks were then placed on a heated pad at dorsal recumbency, and an incision was made to open the skin and the muscle, exposing the abdominal cavity. The yolk sac located at the bottom of the body cavity was removed from the abdomen by ligating (to stop the blood flow) and cutting the blood supply below the ligation (yolk sac side). The abdominal viscera were gently moved to allow exposure and removal of the host's ovary. Bent forceps were used to remove the ovarian tissue. Donor ovarian tissue was orthotopically implanted into the host chicken. The incision was closed using multiple sutures (Monocryl; Ethicon US). Before recovery from anesthesia, 0.1 to 0.3 mL of subcutaneous saline was administrated to replace fluid body loss and maintain blood pressure. After surgery, chicks were placed in a recovery cage with water and food, equipped with a heating lamp, and given an antibiotic (0.2 mg ceftiofur (Excenel, Zoetis Canada Inc., Kirkland, Canada)) and an immunosuppressant (100 mg/kg of mycophenolate mofetil (Cellcept, Hoffmann-LaRoche Ltd., Mississauga, Canada)). Only one surgeon performed these surgical procedures to minimize variations. The immunosuppressive drug was administered daily for the entire experiment to minimize the chances of tissue rejection. At maturity (5–8 mo of age), artificial insemination was performed on the laying hens that were ovarian transplant hosts, and fertilized eggs were incubated until d 10 to 15. These eggs were cooled for an hour at −20°C, and DNA was extracted from the embryo for genotyping.

### Histological Examination

Histological examination investigated differences in ovarian structure between control and donor-derived ovarian tissues. Hens aged 6 to 8 mo were humanely euthanized to collect ovarian tissues. Five confirmed donor-derived grafts were evaluated by the hematoxylin and eosin (**H&E**) staining technique and compared to 2 control ovaries (without surgery). The integrity of the ovarian structures (cortex and medulla) and the presence of immune cells were parameters considered in this histological evaluation.

Harvested tissues were cleaned to remove the blood and connective tissue and immediately fixed in Bouin's solution for 24 h at 4°C. After fixation, tissues were washed 3 times in 70% ethanol and stored immersed in 70% ethanol at 4°C. Fixed samples were loaded into biopsy embedding cassettes and placed into the tissue processor (Leica ASP300S, Leica Biosystems NusslochGmbH, Nussloch, Germany).

Processed tissues were embedded in wax and sliced using a microtome (Rankin 17040, MI, USA) into 4 µm sections transferred onto slides. After mounting, the slides were dried on a warmer plate overnight to evaporate any potentially trapped water under the tissue section. Slides were stained using the standard H&E protocol (Mayer, 1937). The sections were viewed and photographed using a conventional light microscope at various magnifications.

## RESULTS

### Experiment 1. Transplantation Between Hosts of Similar Genetic Background

The aim of experiment 1 was to determine the tissue acceptance rate of fresh or vitrified gonads in a host of similar genetic backgrounds; results are summarized in Table 1. There were no donor-derived ovarian tissues present in any of the LW host hens. In contrast, graft tissues were detected in 4 of the 7 BR host hens when examined at sexual maturity. Furthermore, one of these BR host hens had functional ovarian tissue and laid 6 fertilized eggs. Genotype testing confirmed that these 6 embryos were derived from the donor ovaries, thus indicating successful transplantation in terms of both function (ability to lay hatching eggs) and genetic source (donor-derived).

**Table 1:** Experiment 1. Transplantation between similar genetic backgrounds.

Ovary donor	Treatment	Recipient	Ovarian grafts derived from donor (n)/total chicks
**LW**	Fresh	**LW**	0/4
**LW**	Vitrified-warmed	**LW**	0/5
**BR**	Fresh	**BR**	2/4
**BR**	Vitrified-warmed	**BR**	2/3

Abbreviations: BR, Barred Rock; LW, Lohmann White.

MHC status (mismatched [LW] or matched [BR]) between the recipient and the donor, the status of the tissue (fresh or vitrified-warmed), and the number of ovarian grafts present at maturity are indicated.

While this experiment had limited sample numbers, the results are as expected based on MHC-B status, with successful ovarian transplantation seen only when the MHC-B is matched. However, the MHC-B match or mismatch status is confounded within the line; thus, we cannot distinguish the impact of the line on the results.

### Experiment 2. Transplantation Between Hosts With Matched or Mismatched MHC

Experiment 2 is the classical transplantation experiment within which the possible combinations of MHC-B type were tested for transplantation acceptance. Two flocks were utilized, each with a single MHC-B haplotype. In addition, F_1_ (BL/BR) progenies, which would contain both MHC-B types, were produced by crossing the 2 pure lines. Transplantation of tissue within each line (i.e. BR to BR or BL to BL) would have the identity of MHC-B types and thus be expected to show no MHC-B induced graft rejection. Table 2 shows the transplantation combinations and summarizes both the expected and observed results. There were a total of 43 host hens in this experiment, of which 11 produced eggs for embryo production and testing. However, none of these layer hen's ovaries were donor-derived. The presence of graft tissue was identified in 10 host hens, 6 of which also showed the presence of a host ovary. Within the subgroups expected to reject the transplanted ovary, graft tissue was identified in 4 out of 19 hens, with one hen having the graft co-localized with the recipient ovary. For the subgroups expected to accept the transplanted ovary, we observed 6 out of 24 hens with donor-derived tissue, with 5 of the hens having the graft co-localized with the remnant host ovary. Note that BR/BR hosts had no confirmed grafts irregardless of the genetic background of the donor.

**Table 2:** Presence of ovarian grafts in hosts of similar or different genetic background.

Abbreviations: BL, Brown Leghorn; BL/BR, Brown Leghorn mixed Barred Plymouth Rock; BR, Barred Plymouth Rock.

Perfect MHC-B match, the host should accept the graft.

Mis: MHC-B mismatch, the host should reject the graft.

(# co-localized) = Positive graft was co-localized with a confirmed remnant ovary of the host.

^1^Number of hosts laying fertilized eggs from the donor-derived ovary.

^2^Number of hosts having one or more ovarian tissues to harvest at autopsy.

^3^Number of hosts having a donor-derived ovarian tissue.

^4^Number of confirmed grafts co-localized with a remnant ovary derived from the host.

### Necropsy Assessment of Adult Hens

**Figure 1:**
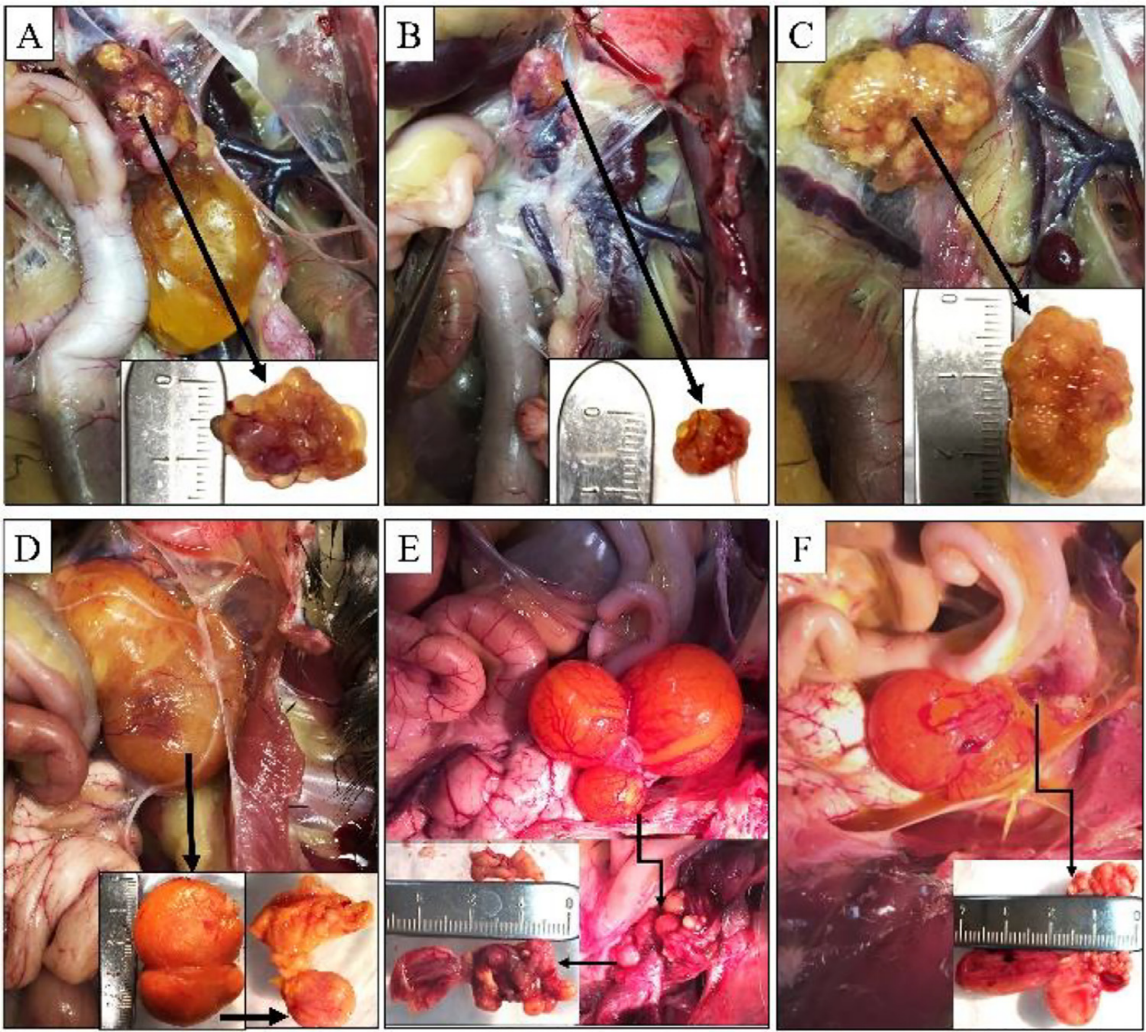
Necropsy assessment of adult hens. Grafts were covered with membranes filled with fluid (A–D) or located at the bottom of the host's ovaries (E, F) showed inside the opened abdomen cavity and detached membranes with fluid (bottom corner of pictures). The size and shape of ovarian tissues varied from hen to hen (ranging from 0.5 to 4 cm).

Donor ovarian tissues recovered from hosts showed various sizes and shapes (ranging from 0.5–4 cm; data not shown). The morphological evaluation revealed that 6 of the 10 confirmed grafts were encapsulated by a fluid-filled membrane (Figures 1A–1D). In the remaining 4 hens, grafts were located at the bottom of the host's ovaries remnant. A thin membrane had formed to separate the grafts and the host's ovaries. These grafts were smaller than the host's ovaries and appeared to have no biological function (Figures 1E and 1F).

### Histological Assessment

**Figure 2:**
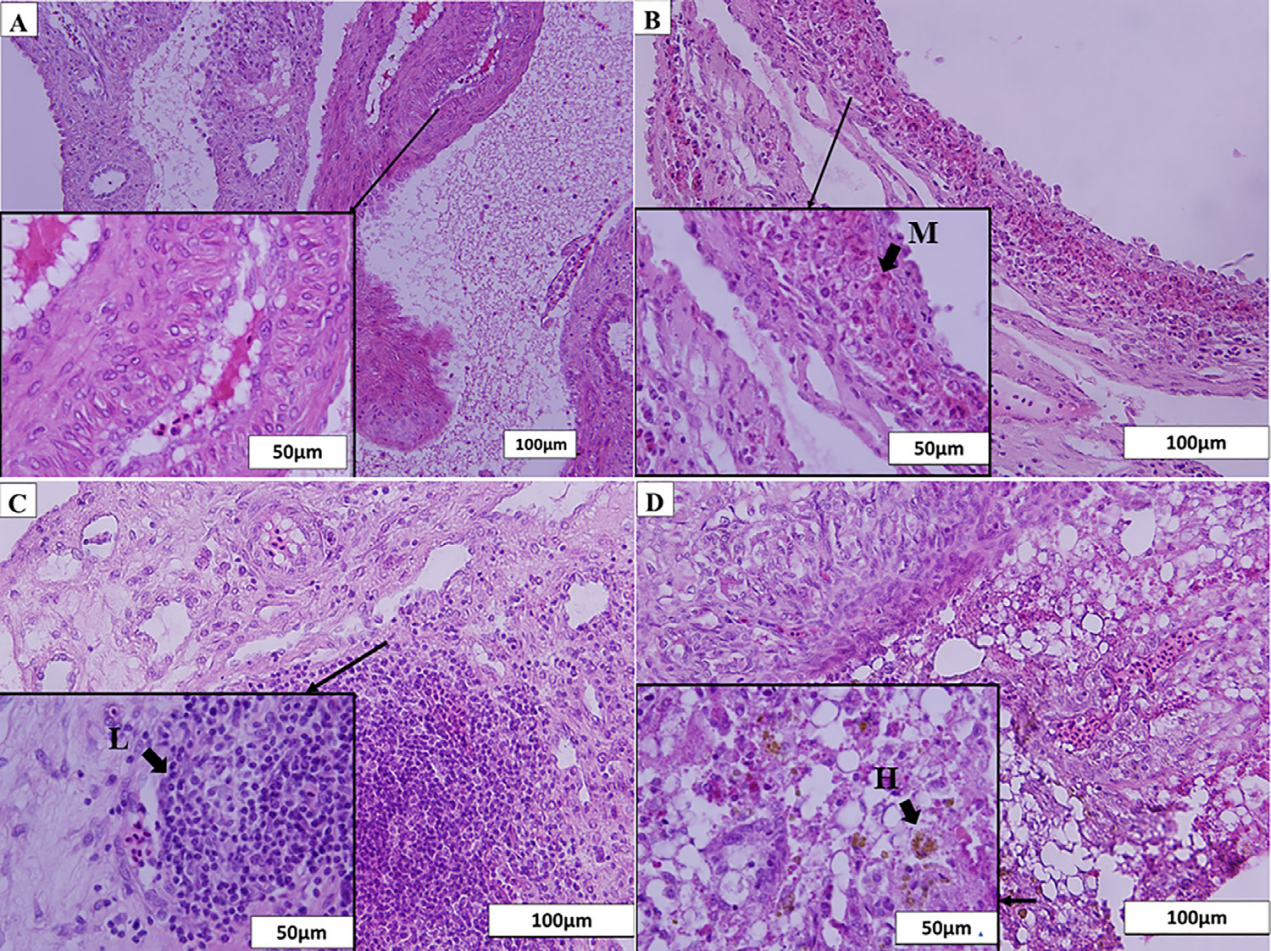
H&E-stained ovarian grafts. H&E-stained ovarian grafts showed a sign of inflammatory response with macrophages (M), lymphocytes (L), and heterophils (H) at the left corner with arrows (1,000× magnification) in images (B), (C), and (D), respectively at 400× magnification. Normal histological morphology of the control ovary showed in image (A) at 200× magnification.

Histological inspection of ovarian tissue from control hens clearly showed cortex cells stained with hematoxylin (dark blue) and medulla in eosin dye (bright pink), with the vascular medulla enveloped by the outer cortex (Figure 2A). In comparison, the cortex and medulla of donor-derived ovarian tissues were not distinguishable (Figures 2B–2D). Immune cells within the ovaries of control birds were scant in the cortex and the medulla of the histological section of the control ovaries (Figure 2A). Donor-derived ovarian tissues showed infiltration of immune cells in both areas by histological evidence of inflammatory responses (Figures 2B and 2C). Heterophil infiltrates (H) were detected in the ovarian cortex (Figure 2B), whereas lymphocyte (L) infiltrates were observed in the cortex and the medulla (Figure 2C). Golden-brown hemosiderin (contained in macrophages) was also observed (M) (Figure 2D). In summary, histological sections of donor ovaries showed clusters of immune cells distributed over the cortex and the medulla of the grafts, but these immune cells were scant in nontransplanted ovaries.

## DISCUSSION

The purpose of this study was to gain insights into the fate of transplanted ovarian tissue, and in particular, to examine the impact of the MHC-B on graft survival. We pose several questions to place our results in the context of the ovarian transplantation technique and MHC-B involvement with tissue rejection.

### Surgical Technique and Fresh or Vitrified Tissue

Transfer of gonads into a host is a technique requiring high-precision surgical skills, specialized equipment, and daily monitoring of postsurgical birds. In our study, only one surgeon performed the surgical procedures to minimize technical variations. Few groups have reported success in regenerating poultry genetics using this technique (Song and Silversides, 2008; Liptoi et al., 2013, 2020; Hall et al., 2022b). These studies used different breeds as a host, which generated various results on the growth of the donor graft. In our study, we observed morphological differences in the vascularization of the yolk sack between our breeds (data not shown). The BR birds had a high vascularization and thinner yolk sack membrane compared to other breeds used in our study. So, the yolk sack easily ruptured while pulling it out, leading to leakage of the yolk into the abdominal cavity and excessive bleeding due to several tears of blood vessels around it. In general, excessive bleeding was the main cause of mortality after surgery, as reported in other studies (Liptoi et al., 2020; Hall et al., 2022b; Santiago-Moreno and Blesbois, 2022). The 1- to 3-day-old ovary of the bird is sitting next to the caudal vena cava and the iliac vein; the abdominal aorta is also in proximity (Song and Silversides, 2006). The initial number of recipient birds utilized for surgery was 70; however, our survival rate was only 61% due to high mortality during or within the first month postsurgery. The mortality rate caused by excess bleeding during the surgery was higher in BR/BR birds (7 out of 21 total birds) compared to BL/BL (3 out of 27 total birds) or BL/BR (0 out of 22 total recipients). This suggests that the physiology of specific breeds involved in the surgery, both donor and recipient, can impact a successful surgery and transplantation. It explains the low number of birds reported in this study.

We also tested the success rate of both fresh and vitrified tissue to sustain growth in a host. Within the BR line, which all had the identical MHC-B type, 4 of the 7 hosts showed sustained growth of either fresh (2 out of 4 recipients; and one of them being functional) or vitrified-warmed ovarian graft (2 out of 3 recipients; Table 1), demonstrating that we could successfully transfer both types of tissue. These positive results demonstrated that our team could potentially regenerate the genetics of a poultry breed using the gonadal transfer technique.

### Is MHC-B Involved in a Recipient's Immune Response Against an Ovarian Graft?

For the first experiment, the LW birds were chosen such that the MHC-B types differed between donor and host, so graft survival was not expected due to MHC incompatibility. All 9 hosts failed to sustain a growth of fresh or vitrified-warmed ovarian graft (Table 1). In contrast, the BR birds had a fixed MHC-B haplotype (Fulton et al., 2016), and graft survival was expected due to a perfect MHC-B match between donor and host. More than half of the BR hosts (4/7) sustained growth of fresh or vitrified-warmed ovarian grafts. The observations from the first experiment suggest that matched MHC-B haplotype may be important to ensure the growth of an ovarian graft into a host; however, the number of birds used for experiment 1 is too low to be conclusive.

Experiment 2 is a classical experiment on transferring a foreign tissue into a host of similar or different MHC-B type. The donors and hosts from BR and BL flocks were selected to have only one fixed MHC-B allele (BR was BSNP-P04, and BL was BSNP-O01; Fulton et al., 2016) with the F1 cross having one copy of each MHC-B alleles (BL/BR) found in the parent. Thus, transferred gonads have a perfect MHC-B match with each line or from parent to F1 progeny (BL/BL to BL/BL, BL/BL to BL/BR, BR/BR to BR/BL, and BR/BR to BR/BR). Gonadal transplantation between lines or from F1 progeny to either parent would have an MHC-B mismatch (BL/BL to BR/BR, BR/BR to BL/BL, BL/BR to BL/BL, and BL/BR to BR/BR). Surprisingly, only 6 donor grafts were recovered from the 24 hosts (success rate of 25%) when a perfect MHC-B match was present between the donor and the host. With an MHC-B mismatch, 4 out of 19 hosts had a graft at maturity (21% success rate). These numbers are in the low range of some results reported by the group of Liptoi et al. (2013, 2020) or similar to Kosenko’ study (2007; used fresh ovaries), but considerably lower than the results reported by Song and Silversides (2008; used fresh ovaries). The presence of ovarian growth with host and donor MHC-B mismatch was unexpected. However, all ovarian grafts from all crosses were small and nonfunctional (Figure 1) regardless of MHC-B match status. Clusters of immune cells were found in the cortex and medulla of all ovarian grafts, including those with MHC-B match of host and donor (Figure 2). Infiltration of immune cells in turkey ovarian grafts was reported in turkey hosts of the same genetic background 6 d postsurgery (Hall et al., 2022b). Therefore, we can hypothesize that our hosts having no grafts may have had strong immune responses during their growth to reach maturity. In addition to the immune cells infiltration observations, 6 out of 9 ovarian graft tissues were encapsulated with a serous membrane filled with fluid (Figure 2) suggesting a strong immune response by the host. Other studies have also reported this encapsulation phenomenon in hosts (Kosenko, 2007; Liptoi et al., 2020). In summary, our results suggest that our recipients kept their immune response against the donor grafts regardless of the genetic background, including a matched MHC-B allele between host and donor.

### Could the Immunosuppressive Agent Have Affected the Immune Response of the Host?

The presence of immune cells in the graft indicates that the immunosuppressive agent did not completely block the proliferation of T- and B- Cells (Figure 2). Mycophenolate mofetil was used daily to suppress the host's immune response for up to 32 wk of this study to overcome the rejection of the donor. In contrast, other studies using the same immunosuppressant at the same concentration had a limited time of immune suppression (a total of 8 wk [daily for the first 2 wk and once a week for the following 6 wk] and a concentration of 100 mg/kg; Song and Silversides, 2007a; Liptoi et al., 2013, 2020), and 2 studies did not use any immunosuppressant drugs (Kosenko, 2007; Song and Silversides, 2007b). These different studies obtained various graft regeneration successes from 0% to 100%. Genetic compatibility between donor and host was suggested as the main factor for having a vitrified-warmed ovarian tissue to sustain constant growth and to promote immune tolerance by the host (Liptoi et al., 2013, 2020). Mycophenolate mofetil is an inhibitor of T- and B-cell proliferation (Morris et al., 1990), and it is used in many protocols to suppress the rejection of organs for human transplant. Thus, the host's immune response should have been minimal if T-cells and B-cell activation were truly suppressed. Unfortunately, 33 matured hosts did not have an ovarian graft (only 10 hosts had a confirmed graft; Table 2), suggesting a strong immune response by these hosts. It is possible that a significant portion of the graft could not implant adequately after the surgery. However, our previous preliminary experiments demonstrated implantation of the vitrified-warmed tissues for most hosts in the first 8 wk postsurgery (data not shown). In addition, the other studies demonstrated proper graft vascularization in chicken (Song and Silversides, 2006) and turkey (Hall et al., 2022b). So, rejection of the graft by an immune response of the host seems to be the major factor causing this low success of ovarian graft growth in a recipient. Our rate of ovarian growth in this study was similar to a study that did not use an immunosuppressive drug (Kosenko, 2007). In addition, it was demonstrated that nonimmunosuppressed hosts could produce progeny derived from a donor-ovarian graft (Song and Silversides, 2007b). The same inhibitor of T- and B-cell proliferation was used to evaluate its efficiency in suppressing the immune response of a turkey recipient against a turkey ovarian graft of similar background (Hall et al., 2022a). This immunosuppressor, used at the same concentration in a turkey host, could not block the infiltration of immune cells into the graft. Our results confirm that 100 mg per kg of mycophenolate mofetil concentration does not adequately interfere with the host immune response in poultry breeds. However, a dose-response study would determine if this immunosuppressive agent can have a repressive response on the immune system of a poultry host.

### Could Vitrifying and Warming Procedures Affect the Ability of Ovarian Tissue to Sustain Growth in a Host?

Processes to store poultry ovarian tissues in liquid nitrogen affect the integrity of the cellular organization of ovarian tissues in birds (Hall et al., 2020), which could reduce the ability of the vitrified-warmed ovarian tissue to sustain growth in a host. In our study, cortex and medulla structures could not be distinguished in donor ovarian tissue recovered from mature hosts (Figure 2). In addition, the full functionality of an ovarian graft could only be obtained using a fresh ovarian graft (Table 1). However, vitrified-warmed ovarian grafts kept growing in some hosts until maturity; confirmed grafts were found in 10 out of 43 hosts (Table 2; only vitrified-warmed ovaries were used in this experiment). The growth of vitrified-warmed gonadal tissue in a host was also reported by Liptoi's group (Liptoi et al., 2020). Harvested grafts in our study had many clusters of immune cells and were not functional (Figures 1 and 2), suggesting that the immune response by the host was the main factor for the low survival of the ovarian tissue. Regardless of the immune response factor, optimizing vitrification-warming procedures should increase the ability of the ovarian tissue to sustain growth into a host.

### Can the Presence of a Remnant Ovary of a Host Influence the Growth of a Graft?

Most recovered ovarian tissues were of host origin (39 out of 43; Table 2), suggesting the ablation of the host's ovary at surgery was not optimal for our study. Having the ovary sitting next to the *iliac communis* vein challenges the surgeon to remove the recipient's gonad completely without excessive bleeding (Song and Silversides, 2006; Liptoi et al., 2020; Hall et al., 2022b). In our study, 60% to 90% of the host's ovary was removed using forceps during surgery (data not shown). Many small remnants ovary could be harvested at the necropsy of a host (Supplemental Data 1). It was suggested that the electrocautery technique might be more suitable for removing the host's ovary; however, this technique still does not completely ablate the whole ovarian tissue (Liptoi et al., 2020). Thus, the transferred graft would grow next to the host's remnant ovary, and the presence of the host's remnant ovarian tissue may negatively influence the development of the ovarian graft. Interestingly, the presence of a remnant turkey graft did not influence the implementation and the growth of a turkey ovarian graft 6 d postsurgery (Hall et al., 2022b). In contrast, gonadectomy of the host testes is done to improve graft growth in xenotransplantation studies (Schlatt et al., 2003; Rathi et al., 2006). Another study did not find a difference in the xenograft growth of a recipient mouse when its testes were present (Abbasi and Honaramooz, 2011), but the ovarian tissue may respond differently. The graft size was generally smaller than the co-localized remnant host ovary, but the number of positive grafts was too low to make conclusions (data not shown). In addition, some of the remnant host ovaries were functional, and the recipient could lay eggs (11 out of 43 recipients; Table 2). More studies are required to determine if the remnant chicken ovary in a host could negatively influence the growth of an ovarian graft.

### Should Ovarian Tissue Be Preserved in Liquid Nitrogen if the Regeneration Procedure Is Not Optimal?

If the transfer of gonad protocol needs to be optimized to sustain growth in a host, vitrification-warming procedures can still be used to preserve gonadal tissue from important commercial or heritage breeds. In our study, vitrified-warmed ovarian tissues were still present at maturity in hosts of similar or different genetic backgrounds (Tables 1 and 2). These ovarian tissues were preserved in liquid nitrogen for at least 5 d before their usage, and many of them were stored for months in cryotanks. Other studies using vitrified-warmed poultry ovarian tissues reported similar observations (Hall et al., 2020; Liptoi et al., 2020). Therefore, the vitrified gonadal tissue is still viable after warming. Animal gene bank managers can use the vitrification of poultry ovarian tissues to preserve heritage breeds classified as critical or endangered by many countries, regardless of the low regeneration of a poultry breed reported by several studies when using the gonadal transfer technique (Kosenko, 2007; Liptoi et al., 2020). If a few birds can be produced using the gonadal transfer technique with vitrified-warmed gonads, it would still be sufficient to develop strategies to regenerate a poultry breed. However, we cannot conclude that vitrification of ovarian tissue is a “viable” cryopreservation method until it is shown to work for multiple breeds and in multiple laboratories. So, ovarian tissue integrity after vitrification should be analyzed to determine its viability. Also, factors influencing the genetic compatibility between donor and recipient need to be identified, which could reduce the variability of the success rate reported by several studies (Kosenko, 2007; Liptoi et al., 2013, 2020). Therefore, continuing research on improving existing methods or developing additional methods is critically important for preserving rare and endangered breeds and their genetic variability.

## Appendix Supplementary materials

Image, application 1

## Footnotes

Supplementary material associated with this article can be found in the online version at doi:10.1016/j.psj.2023.102850.
